# They don’t want to close Roxy: a qualitative account on the perceived efforts by Ivory Coast to end the informal market for medicines

**DOI:** 10.1016/j.hpopen.2025.100145

**Published:** 2025-07-22

**Authors:** Victor Chimhutu, Armel Dagrou, Archlove Takunda Tanyanyiwa

**Affiliations:** a Department of Public Health and Sports Sciences, Inland Norway University of Applied Sciences, Postbox 400, 2418 Elverum, Norway; b Department of Health Promotion and Development, University of Bergen, Postboks 7807, 5020 Bergen, Norway; c Department of Social Work and Guidance, Inland Norway University of Applied Sciences, Vormstuguvegen 2, 2624 Lillehammer, Norway

**Keywords:** Pharmaceutical drugs, Sub-saharan africa, Informal markets, Informal medicines, Informal providers

## Abstract

The informal market for medicines poses great danger to public health as they expose populations to counterfeit and expired medicines, among many vices. Ivory Coast is one of the countries where this market is growing. The country has been trying to end this market unsuccessfully. This study aims to investigate the perceived role of the state in the regulation and efforts to end this market. A qualitative case study design was used, with in-depth interviews (IDIs) and focus group discussions (FGDs) being the methods for data collection. 20 IDIs and 3 FGDs with 13 participants in total were conducted with sellers, buyers, and pharmaceutical experts. We found that: the informal market plays an integral role in the health system and that it provides employment to many. Additionally, this informal market is complex and requires a lot of cooperation and coordination at many levels to successfully end it, which is resource demanding. Based on some of these reasons, there is an ambivalence in the regulation and the quest to end this market. The study concludes that the government of Ivory Coast needs to be decisive in its roles of regulation and that of ensuring that medicines are available and accessible.

## Highlights

• Informal markets for medicines pose a great danger to public health.
• Efforts to regulate and stop the informal markets for medicines are falling short.
• The government is failing in its role in making medicines accessible and available.
• Failure by the state to provide medicines opens an opportunity for informal markets.
• The government need to be decisive in its roles of provision and regulation.

## 1 Introduction

The informal market for medicines poses some risk to public health, which includes poor prescription practices and higher chance of exposure to expired, substandard and even falsified medicine, and yet this market also plays a significant role in ensuring access to medicine in resource constrained environments [1]. This market which is fueled by substandard and falsified medicines has been growing from being a US$80bn to a US$200bn industry between 2010 and 2020 globally [2]. Africa and Asia are the main targets for this industry, with up to 42 % of all these products ending up in the Africa region [1,3,4,5]. Sub-Saharan Africa imports 90 % of its medical products, making it prone to substandard and falsified medical products [1,5]. While this article focuses on Ivory Coast, a country in the sub-Saharan region, it must be emphasized that falsified and substandard medicines are a global phenomenon and challenge [1]. Regulatory authorities globally and especially in resource constrained environments find dealing with informal markets challenging as access to medicines through formal channels is not guaranteed [6] Additionally, measures and regulatory frameworks for the informal market require intersectional, multisectoral and intergovernmental efforts [6], something not easy to achieve but beneficial. As a result of these various challenges, in fragile health systems, informal markets for medicines thrive. Their thriving, is however, worrying especially in this post pandemic period, and in the context where during COVID-19, fake vaccines were reported and confiscated in this region [7,8,9].

In the Africa region, especially French- speaking countries, the selling of pharmaceutical drugs on the informal market is not a new phenomenon [10,11], and Ivory Coast is among these countries where the phenomenon is widespread [12]. This study therefore attempts to contribute to this well-known phenomenon causing rife in the region [13], and as a result contribute to needless mortality [1]. To achieve this, we aim to investigate how the role of the state in the regulation of, and efforts to end the informal market for medicines is perceived by users, sellers, and experts in the pharmaceutical industry.

## 2 Theoretical framework

The informal market for medicines is expanding in Ivory Coast despite various policies and measures to curtail it. An understanding of why policy measures may fail to achieve intended objectives is therefore significant for this study. A framework on policy failure, outlining four broad contributors [14], will be used. Firstly, policies fail because of overly optimistic expectations. When policies are formulated, there is usually an underestimation of resources required and an overestimation of the benefits [14]. An underestimation of resources required for the implementation mean policies will have modest success or fail in toto. Secondly, the implementation is usually in a dispersed governance [14], and this means expectations at national and local levels can vary a lot. National level policies usually do not consider local conditions and contexts, and as a result this can contribute to policy failure. Thirdly, a lack of collaborative policymaking can lead to policy failure [14]. Policymakers tend to operate in administrative silos and forget that the policies they are making will have wider consequences outside of the targeted sectors. In instances where collaborative policymaking exists, policies may still fail because different actors involved usually do not establish a common ground for public problem solving [15]. Implementation normally has unforeseen challenges, therefore this common ground for problem solving is important. Lastly, policies do fail due to vagaries of political cycles [14]. Politicians operate with electoral cycles, that is with votes in mind. A politician is unlikely to come up with a decisive policy action if that action may cost him or her votes. In this regard, politicians may prefer piece-meal reforms to an existing challenge for short term results than a substantial reform that may sacrifice their popularity.

These four dimensions will be useful in this study to shed light on accounts from our research participants based on their experiences and privileged positions with regard to government action or inaction on the informal market for medicines. While this framework helps to analyze why policy or policy action fail, it does not assign any responsibility to the state, which is important in the context of this study, as the informal market for medicines is largely in place because states fail in their duties to ensure that medicines are available in right quantities and are accessible for the citizens. To cover this dimension, we will use the AAAQ model (Availability, Accessibility, Acceptability and Quality) [16].

This framework comes from a rights perspective and has been used in health-related research that deals with provision, access and utilization of health services and products [17]. As health is a human right, it is the state’s responsibility to make sure that health services and basic medical products are available, accessible, acceptable and that they are of good quality.

## 3 Methods

### 3.1 Study design

A qualitative case study design was chosen for this study. The case that was used in this study is of Roxy, one of the most popular informal markets for medicines Ivory Coast and west Africa. The study targeted three groups of participants, the sellers and buyers, and the pharmaceutical experts. The pharmaceutical experts were selected based on their vast knowledge on the pharmaceutical industry, and the regulatory frameworks of this industry in Ivory Coast. Purposive selection of participants was therefore used in this study.

This triangulation of the study participants provided us with rich data, which improved the quality and credibility of the study. Recruiting the sellers and buyers was challenging in the beginning, given that the informal market for medicines is considered an illegal market. Mistrust was, therefore, the main challenge initially, especially in recruiting sellers. However, we then used our social networks in the context and snowballing technique for recruitment. Building rapport with participants, was significant for this study to establish trust. The recruitment of pharmaceutical experts was not challenging as the 2nd author was doing an internship at one of the leading institutions that deals with issues around public health and regulation of the informal market for medicines at the time of the fieldwork.

### 3.2 Data collection

Data collection for the study was done in two phases, in January of 2019 and from December 2019- January 2020. Data that was collected for the first phase was mainly used in a master thesis of the 2nd co-author [18]. However, after realizing the richness of the data and its importance in the field, we then decided to collect additional data. Additional data was also collected because after data analysis for the master thesis, we felt that data saturation was not reached, as a result, additional data collection helped with exploring some of the themes and aspects we felt were not exhaustively addressed by the scope of the master thesis. We, however, cannot claim that data saturation was reached but additional data collection helped in this direction. In-depth interviews (IDIs) and focus group discussions (FGDs) were used for data collection. 20 IDIs were conducted in total, six with pharmaceutical experts and fourteen with sellers. Table 1 gives an overview of the IDIs.

**Table 1:** Overview of IDIs with pharmaceutical experts and sellers.

**Category of participants**	**IDIs 1st Phase**	**IDIs 2nd Phase**	**Total**
Sellers	7	7	14
Pharmaceutical experts	2	4	6
**TOTAL**	**9**	**11**	**20**

A total of three FGDs were conducted with the buyers, and two of these were done during the first phase of data collection and one during the second phase. The three FGDs had thirteen participants, four women and nine men. Participants were not separated by gender during the FGDs as the topics discussed were not gender sensitive. Table 2 gives an overview of FGDs.

**Table 2:** Overview of FGDs with buyers/users.

**FGD number**	**Women**	**Men**	**Total**
FGD 1	0	5	5
FGD 2	2	2	4
FGD 3	2	2	4
**TOTAL**	**4**	**9**	**13**

All IDIs and FGDs were conducted in French, which is the official language of Ivory Coast. The interview and topic guides covered the following thematic areas: experiences with and perceptions of the informal drug market, and regulatory frameworks and policy action targeting the informal market for medicines. Data was collected by the 2nd author of this article, who is a citizen of Ivory Coast but was a master student in Norway at the time. Being a citizen of Ivory Coast helped during data collection to create rapport with participants, it also makes it easy as the 2nd author had linguistic competence. In other words, the 2nd author was an insider most of the time during data collection.

### 3.3 Analysis

All IDIs and FGDs were voice-recorded, after permission was sought for and granted by the participants. After the data collection process, data was transcribed in French and then translated to English. After this stage the analysis of data began in NVivo 12, using thematic analysis [19]. The transcripts were subjected to a thorough review before the coding exercise began. Themes that emerged during this process were used to present the results of this study.

### 3.4 Research ethics

Ethical clearance was granted in Norway by the Norwegian Centre for Research Data (NSD now SIKT) and in Ivory Coast permission was granted to conduct the study by the Directorate for Pharmacies, Drugs and Laboratories (DPDL). Participation was voluntary. Most participants gave written consent while in few cases consent was given orally. Confidentiality and anonymity were assured. We have depersonalized data by giving research participants pseudo-names. Pharmaceutical experts are also not identified by their organizations or real names in this study.

## 4 Results

This section presents the study findings. The three categories of our research participants put the measures by the government to combat the informal market for medicines under scrutiny. The four themes that will be presented are complementary and at times conflicting. Some of our participants saw the government to be doing everything possible to curtail the informal market, however, this was heavily contested by others. Some sympathised with the role of the government, while others accused the powerful people of fuelling the market.

### 4.1 The government is trying to stop the informal market

Julien, one of the pharmaceutical experts feels the government is doing all it can to fight the informal market. The issue of medical drugs and products on the informal market is a public health challenge to which the government is responding by establishing appropriate structures:

*“The fight against illicit trafficking of drugs here in Ivory Coast is organized by the COTRAMED (National Committee to Combat Illicit Trafficking and Counterfeit Medicines). It is an interdepartmental committee whose activities are essentially to eradicate the informal drug market. It is an inter-ministerial committee and is composed of the Ministry of Internal Affairs, represented by the police, especially the police for narcotics and drugs, the Ministry of Defense represented by the national gendarmerie and its anti-drug section, the Ministry of Justice, the Ministry of Communication and finally the Ministry of Health. They all are working together to tackle this issue, you see how the government is really trying to eradicate this challenge by setting up such a highly powered structure”* (Julien, Pharmaceutical Expert- IDI)

Lysette, another pharmaceutical expert also shared the same view:

*“You know the government is really trying, COTRAMED is trying, I think you know that they carry out many raids at Roxy, but the market comes up again and again. Some of these operations seize huge quantities of drugs, most of it is fake medicines. You see how big the problem is?”* (Lysette, Pharmaceutical Expert- IDI)

In addition to regulatory framework and its enforcements through raids, the government has also put in place other strategies to combat the informal market. For example, the campaigns sensitizing the population about the risks linked to the consumption of drugs from the informal market. These campaigns are done through various mediums of communication and are done through the Directorate for Pharmacies and Medicinal Products (DPMP). These campaigns include television programs and advertisements informing people about the risks linked to the drugs sold on the informal market such as Roxy:

*“Last time, the DPMP was live on the national television and took part in a discussion with consumers from the informal market about this issue. We are even trying to set up platform on WhatsApp so that everyone could be able to exchange a little bit on this topic and share experiences.”* (Julien, Pharmaceutical Expert- IDI)

In addition to television advertisements and programs, there are also other various awareness campaigns aimed at discouraging people from consuming drugs from the informal market for pharmaceutical drugs:

“*There have been awareness campaigns against the street sales of drugs such as “my health, my life” or “my health first” among others. But maybe, these campaigns do not carry much because until now this phenomenon has been persisting.”* (Frederic, Pharmaceutical Expert- IDI)

According to Frederic, billboards awareness campaigns were the most effective, there was one which was able to reach and appeal to many people, clearly outlining the dangers of drugs from the informal market.

**Fig. 1:**
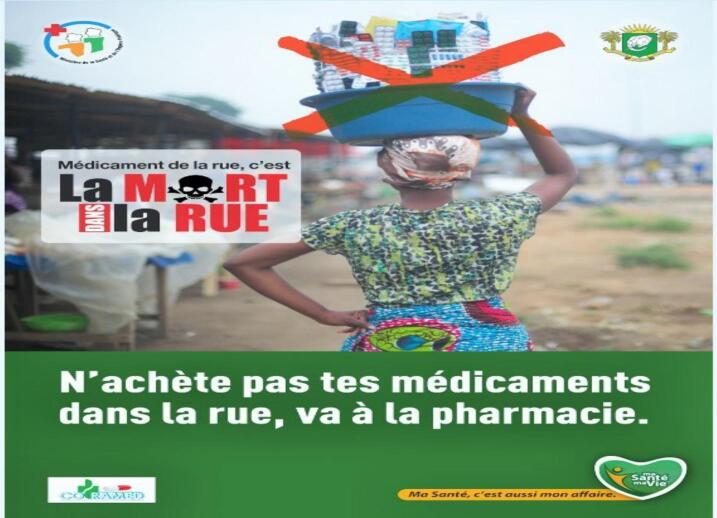
(source- billboard fieldwork photo).

The advert has the following words (see Fig. 1), *“pharmaceutical drugs from the street leads to death, do not buy your drugs in the street, go to the drugstore.”* (own translation from French)

Raymond, another pharmaceutical expert, did not share the same view with Julien, Lysette and Frederic on the effectiveness of the awareness campaigns. He pointed to the fact that the IMPD was thriving, hence difficult to argue for effectiveness of campaigns:

*“Awareness campaigns do not work because people are used to these products and do not see the importance of buying drugs at the pharmacy. In my opinion we should also punish people caught up in the process of buying drugs in unofficial drugstores”* (Raymond, Pharmaceutical Expert- IDI)

While critiquing the awareness campaigns, Raymond here raised an important point. Laws available in Ivory Coast on activities on the informal market are silent on buyers, the users, but sanction sellers. In other words, it is legal to buy medical products at the informal market but illegal to sell such drugs. When demand is high, it is difficult to stop the market.

### 4.2 The government is not doing enough to stop the informal market

While many of the pharmaceutical experts were of the opinion that the government is doing all it can to end the informal market, Raymond and other participants did not share this view. Raymond was adamant that the government was not doing enough and questioned its sincerity:

*“I find it hard to believe that the government really wants Roxy market to disappear. In Adjamé there is a police station less than 500 m away from Roxy market and they try to make us believe that they are fighting this market? Do you think it is fair that people sell death without being bothered by the police?* (Raymond, Pharmaceutical Expert- IDI)

Raymond went further to suggest that maybe the government inaction or lack of decisive action was because they were misjudging and underestimating the public health threat:

*“The informal sale of pharmaceutical drugs is a serious threat to Ivory Coast, and I feel like the higher authorities do not have a real idea of the impact that this has on the health of populations. You will see, in a few years the government will have to create hundreds of drug rehabilitation centers for these young people who are taking these drugs for fun.”* (Raymond, Pharmaceutical Expert- IDI)

Here, Raymond is also alluding to the fact that the informal market makes dangerous or addictive medical products easily available without prescriptions and this leads to the serious public health problem of addiction especially among young people. Users who participated in FGDs also noted that the government was probably not doing enough to fight the informal drug market maybe not because they are underestimating its threat as Raymond puts it above but because the government has other pressing issues:

*“The government has bigger issues to tackle at this moment. Right now, the farmers are complaining about the price of Cocoa. Between this you think the government can focus on Roxy. These small traders, I don’t this so, these small traders aren’t bothering them.”* (Serge, Buyer – FGD 1)

Paul another participant also added that he feels the government has other important issues and using scarce resources on stopping the informal market for medicines is tantamount to wasting resources. Additionally, Roxy provides employment for many people, and this on its own may reduce the government’s political will to take on the market decisively:

*“Do you think the government is going to waste money by deploying people on the field to deal with Roxy? Afterall Roxy is employing many people, will the government create employment for these vendors? I don’t think so”*. (Paul, Buyer- FGD 1)

This view by Paul was also widely echoed by sellers at Roxy, the market is a source of livelihood for them. They are aware that it is not a stable and predictable source of income, due to constant raids by the authorities, however, it is the only job they have. Many of them started selling drugs when they were very young hence the only skill they have:

*“Selling drugs is the only proper job I know, I started doing this when I was very young. Many members in our family do this, so it was easy to learn. The police want us to stop but I do not have another job, my family live from here. My brother, I don’t have an education, what other job can I do? If they want us to stop then they must give me another job”*. (Helene, Seller- IDI)

Many sellers fundamentally agree with this view from Helene, many of them started selling at Roxy from a young age, some as young as six years. They also have little or no education, sellers like Assia for example told us that when she started, she could not even know how to count money neither the names of the medicines she was selling. However, Assia reckons that the government has the power to stop them if it really wants to, but this was also a political matter:

*The government is big and has many resources, Roxy is like a fly. They can crash us if they want. We cannot do much you know to fight back, how can we fight the police, the soldiers, no we cannot do that. But what I know is that this is my food, I eat from here. These people come to us and ask for votes, if they want my vote, how can they close Roxy? Who will vote for them? Is it not us?* (Assia, Seller- IDI)

Assia is raising an important matter here that being a voter gives them some leverage. The government and more precisely the politicians may not act decisively because the informal market not only does it employ many people but also serve many people. Aminata had this to say:

*“Roxy helps us the poor, when we are sick, we buy medicines there, those from Cocody (rich neighbourhood) do not need Roxy, they buy from pharmacies, but us we want Roxy because we cannot buy from pharmacies. This is how it is, and the government knows that. It is not our choice that we are poor. It is not our choice that we cannot afford medicines from the pharmacies. If I want to buy only 2 tablets for headache, the pharmacy cannot give me that. I do not have money to buy the whole pack. The government must be happy we have Roxy, otherwise we could die.”* (Aminata, Buyer – FGD 2)

Aminata who participated in this research as a buyer from Roxy but also sells medical products at another informal market is highlighting on very significant issues here. She is raising the class issue, Roxy serves the poor and vulnerable.

### 4.3 The informal market is complex, the government is overwhelmed

Another view raised was that the informal market is so complex that the government is overwhelmed on how to successfully tackle it. It was noted that the challenge and threat does not only come from within borders but from outside:

*“Drugs sold illicitly in Ivory Coast come from all over the world. There are mafias from Asia who are delivering these drugs throughout west Africa through Benin and Nigeria. Nigeria remains an important country when it comes to counterfeit drug trafficking. There are also counterfeiters in Benin and Nigeria who manufacture these types of drugs.”* (Julien, Pharmaceutical Expert- IDI)

This view that the informal market is complex and a major hindrance to the fight to end this market was also echoed by Frederic another pharmaceutical expert. He noted that even if Ivory Coast succeeds in supressing the market internally, the threat is still just outside of its borders waiting for an opportunity to knock:

*“This is a complex problem to deal with because it has many fronts. Roxy you are talking about is just a drop in the ocean. Yes, Roxy is the most popular in Abidjan but globally it is nothing. Counterfeits and sub standards are sold here in Ivory Coast and at Roxy, but they are not manufactured here. You cannot solve the problem by only closing the market, you need to know the source and the movement of these drugs too. In this, the government cannot succeed alone, it calls for a lot of cooperation.”* (Frederic, Pharmaceutical Expert, IDI)

The government of Ivory Coast is already working with other regional partners in to tackle this public health challenge. In addition, Ivory Coast also want to participate more at the international stage in trying to curtail the informal market scourge in the country:

*“The government wants to become a signatory to the Medicrime Convention. This will allow us to share experiences with countries that are already signatories, and this will also allow us to extend our field of action.”* (Julien, Pharmaceutical Expert- IDI)

While this is a good step for the government of Ivory Coast to take, one may ask why it is taking the country this long to be part of such important international instruments. Estelle, another pharmaceutical expert was critical about the seemingly easy approach to the challenge of the informal market for medicines by African governments:

*“It is mind boggling, it seems our governments are not yet ready to deal with this. The facts are startling but there is no action. Africa is losing many people due to substandard products. You know 10 % of all medical products in Africa are either substandard or counterfeit? These numbers are frightening. People say they are trying, and I say, yes trying is what they are doing. They need to do more than trying.”* (Estelle, Pharmaceutical Expert- IDI)

Estelle here presents the informal market as a problem of great dimensions warranting attention from authorities. We have heard of activities that the government of Ivory Coast are carrying out through COTRAMED to end the informal market. Julien feels these activities and actions are necessary but financially costly:

*“It is important to note that when drugs are seized, we analyse them and then send them to the crematorium to burn them. We cannot take the risk of burying them because people can go and dig them up and put them back on the market, so we send them to a cement factory where there is an incinerator. But all this has a cost, and it is the responsibility of the government to take care of it.”* (Julien, Pharmaceutical Expert, IDI)

### 4.4 There are big powerful people with big interests in the informal market

During our fieldwork, many of our participants in all categories felt that very powerful people were benefitting from this informal market:

“*It should be known that some people who engage in this kind of practice have a long arm and have managed to avoid justice because they have powerful positions or got connections with those in power and in the courts. As you know this is a multi-billion-dollar industry with powerful people.”* (Raymond, Pharmaceutical Expert- IDI)

This same sentiment was also echoed by Estelle, another pharmaceutical expert. She gave some figures on how big the industry is and has been growing. This she says is tempting for those in powerful positions to get involved in this illicit market:

“*We are talking of an industry that is illegal but huge here, one can wonder if it is not being fuelled by very powerful people. This too can explain a lot of things. The industry is estimated to be worthy over US$150bn, that is a big industry there. It is not far-fetched that even our own powerful people are also into in. The industry is lucrative.*” (Estelle, Pharmaceutical Expert- IDI)

From top level, the corruption can also permeate to lower levels of the society and this may make the efforts to combat the informal market fruitless. Sellers also feel this corruption at street level:

*“It is not easy when they come for raids, we lose a lot. But sometimes you ask yourself, what is better to lose everything or to pay a little and keep your stuff. I prefer to do the latter. Is it good, maybe not but what can we do or what can you do in that situation?”* (Fatou, Seller- IDI)

In this vein, corruption to an extent is fuelling the informal drug market at many various levels. This then diminishes the few efforts by the government to combat this market.

## 5 Discussion

We will use two frameworks to guide our discussion, the AAAQ [16] and the policy failure framework [14]. Ivory Coast, like many low-income countries, is failing in its duty to provide adequate basic medical products to its citizens and for that primary reason the informal market is growing unabated [20]. It must be emphasized that it is first and foremost the responsibility of governments to make sure that basic pharmaceutical drugs are available, accessible and of good quality [16] and failure in this duty can leave the general population with no viable option, besides the informal market, which exposed the population to great risk.

Unstable and rampant shortages of pharmaceutical products have been reported by studies from Ivory Coast [12,21,22]. These studies estimate that the formal market satisfies only 30 % of the population’s needs, which makes the informal market integral. When the informal market for medicines becomes an integral part of the health system, we can then question if the government will have the political will to curtail the market. Our participants questioned whether the government was really for clamping down the market or if these efforts were just cosmetic. Studies from Bangladesh [23], Tanzania [24] and Benin [10] and various other low-income contexts [25,26] have also found out that the informal market plays a critical role in the health system, and this can contribute to the policymakers having an ambivalent attitude [24] towards the informal market for medicines.

Although our study was conducted in the capital city, Abidjan, studies have also reported that the informal market is very critical for rural areas with no access to health facilities and services, populations in these settings sorely rely on the informal market [11,21,27]. Given this central role of the informal market, we can then revisit the fourth factor contributory to policy action failure [14], the vagaries of the political cycle, where politicians are less likely to take decisive action if such action will make them unpopular [14]. For example, shutting down Roxy can be politically challenging only for this reason. Given this, politicians may go for easy short-term fixes while sacrificing policies with sustained long-term impacts [28] as long-term solutions may come with some political costs.

The second discussion point is that it is the responsibility of the state to regulate the pharmaceutical market and curb the informal market. Our findings suggest the failure by the government in this task too. Although COTRAMED can be classified as a modest success [28] to efforts to curb the informal market for medicines, the main challenge of setting up such a complex agency, involving many ministries also comes with problems of coordination [29]. The first contributory factor why policies fail is that policymakers are overly optimistic of success and may forget implementation challenges [14]. Narrations from our participants strongly indicate that government agencies are not being successful in the enforcement of the existing laws and one reason that was repeatedly mentioned was of inadequate resources. Something predictable when using the framework of policy failure [14].

It is also important to note that due to various challenges bordering on resource shortages, it is extremely difficult for authorities to ensure that enforcements are regular. In this study it was noted that regulatory inspections were sporadic, making it extremely difficult to successfully curb the informal market. The same phenomenon was also observed in Tanzania where informal drug sellers revealed that they barely experience regulatory inspection at their stalls [24]. Our participants, the sellers and buyers, were quite aware that they are voters and if any decisive action is taken to close Roxy by politicians at local or national level, it was going to come with a political cost. Sellers and buyers, the citizens know they have this power because they are on this market or using this market because the authorities have failed in their tasks of employment creation and provision of adequate pharmaceutical drugs. In this regard, the vagaries of the political cycle [14] can help to explain why politicians may choose not to be decisive on certain policy issues even if opportunities or the policy window is available [30].

The informal market for medicines, in this regard acts as a valve that reduces pressure on politicians to solve drug shortages and unemployment. While this may benefit the politicians in the short term, in the longer term, the informal market is causing an ever-increasing public health threat which require long term and sustained policy action, something that many politicians may not choose as it can be costly to their political ambitions or legacies.

Additionally, the informal sector for medicines is complex and caused by widespread challenges, which may include primarily the inability of governments to make medicines accessible to their populations [12,21,22]. Governments need to make more efforts in making medicines accessible to their populations, any effort in this direction is a movement is solving this challenge. A recent study by Hasnida and colleagues in Indonesia, found that intersectional collaboration and engagement help medicine regulatory bodies in doing a better job and exposes them to new methodologies in the monitoring and tracking of the quality of medicines [6]. The same also goes when this collaboration is extended to intergovernmental collaborations, as the informal market for medicines is a complex phenomenon which has no respect for national or international borders.

### 5.1 Limitations and strengths

Limitation in the current study involves the study design, data collection and selection of participants. The study was first designed as a project for a master’s thesis with a limited scope, we then had to collect additional data during a second phase. The second phase was disrupted by COVID-19 and this limited the amount of data we could collect during this 2nd phase. Another possible weakness but also a strength is we used purposive sampling, this allowed us to target the research participants with the right information, however, this might introduce some biases in that we excluded other potential participants whose views might have enriched this study. Another potential weakness is that sellers might have provided overly positive and biased responses as this is part of their livelihood.

## 6 Conclusion

This study set out to find out the perceived role being played by the government of Ivory Coast in regulating the informal market for pharmaceutical drugs in Ivory Coast. This role of the government was contested. Some felt the government was doing enough while others felt its actions were piece-meal and cosmetic. What emerged is that the informal market of medicines presents a complex challenge because most of the activities are done outside the borders of Ivory Coast. Domestically, this fight is also not easy because the government cannot provide its people with needed medical products, which creates demand. Additionally, this informal market is a source of livelihood for sellers. We, therefore, conclude that the government need to be decisive in its roles of regulation and that of ensuring that pharmaceutical products are available and accessible.

## CRediT authorship contribution statement

**Victor Chimhutu:** Writing – review & editing, Writing – original draft, Supervision, Project administration, Methodology, Formal analysis, Conceptualization. **Armel Dagrou:** Writing – review & editing, Resources, Project administration, Methodology, Investigation, Formal analysis, Conceptualization. **Archlove Takunda Tanyanyiwa:** Writing – review & editing, Project administration, Methodology, Investigation, Formal analysis.

## Funding

This research received no specific grant from any funding agency in the public, commercial, or not-for-profit sectors.

## Declaration of competing interest

The authors declare that they have no known competing financial interests or personal relationships that could have appeared to influence the work reported in this paper.
